# Socio-economic inequalities in the use of drugs for the treatment of chronic diseases in Italy

**DOI:** 10.1186/s12939-022-01772-8

**Published:** 2022-11-09

**Authors:** A. Di Filippo, S. Perna, A. Pierantozzi, F. Milozzi, F. Fortinguerra, N. Caranci, L. Moro, N. Agabiti, V. Belleudi, G. Cesaroni, A. Nardi, T. Spadea, R. Gnavi, F. Trotta

**Affiliations:** 1 Italian Medicines Agency (AIFA), Via del Tritone 181, 00187 Rome, Italy; 2 Regional Health and Social Care Agency, Emilia-Romagna Region, Bologna, Italy; 3 Department of Epidemiology, Lazio Regional Health Service, Rome, Italy; 4 Epidemiology Unit ASL TO3, Piedmont Region, Turin, Italy

**Keywords:** Socio-economic inequalities, Deprivation index, Drug utilization, Adherence, Persistence, Pharmacoequity

## Abstract

**Background:**

Since the use of medicines is strongly correlated to population health needs, higher drug consumption is expected in socio-economical deprived areas. However, no systematic study investigated the relationship between medications use in the treatment of chronic diseases and the socioeconomic position of patients. The purpose of the study is to provide a description, both at national level and with geographical detail, of the use of medicines, in terms of consumption, adherence and persistence, for the treatment of major chronic diseases in groups of population with different level of socioeconomic position.

**Methods:**

A cross-sectional study design was used to define the “prevalent” users during 2018. A longitudinal cohort study design was performed for each chronic disease in new drug users, in 2018 and the following year. A retrospective population-based study, considering all adult Italian residents (i.e. around 50.7 million people aged ≥ 18 years). Different medications were used as a proxy for underlying chronic diseases: hypertension, dyslipidemia, osteoporosis, diabetes and chronic obstructive pulmonary disease. Only “*chronic*” patients who had at least 2 prescriptions within the same subgroup of drugs or specific medications during the year were selected for the analysis. A multidimensional measures of socio-economic position, declined in a national deprivation index at the municipality level, was used to identify and estimate the relationship with drug use indicators. The medicine consumption rate for each pharmacological category was estimated for prevalent users while adherence and persistence to pharmacologic therapy at 12 months were evaluated for new users.

**Results:**

The results highlighted how the socioeconomic deprivation is strongly correlated with the use of medicines: after adjustment by deprivation index, the drug consumption rates decreased, mainly in the most disadvantaged areas, where consumption levels are on average higher than in other areas. On the other hand, the adherence and persistence indicators did not show the same trend.

**Conclusions:**

This study showed that drug consumption is influenced by the level of deprivation consistently with the distribution of diseases. For this reason, the main levers on which it is necessary to act to reduce disparities in health status are mainly related to prevention. Moreover, it is worth pointing out that the use of a municipal deprivation indicator necessarily generates an ecological bias, however, the experience of the present study, which for the first-time deals with the complex and delicate issue of equity in Italian pharmaceutical assistance, sets the stage for new insights that could overcome the limits.

**Supplementary Information:**

The online version contains supplementary material available at 10.1186/s12939-022-01772-8.

## Key points

**• Question:** Is there a relationship (in terms of consumption, adherence and persistence) between socioeconomic position of people with chronic disease and chronic medications use?

**• Findings:** Socioeconomic position was strongly correlated with the use of medications, coherently with the social gradients in the occurrence of most diseases; the analysis of adherence and persistence among groups of population with different socioeconomic deprivation showed a lack of a univocal gradient among the Italian regions.

**• Meaning:** It is necessary to improve prevention of chronic diseases to reduce disparities in health status.

## Introduction

Health inequalities associated with socio-economic position (SEP) have been observed in all European countries [1]. Several studies published over the last 20 years showed that people with a higher social and economic position have better health status. Since available evidence showed that health disparities can be reduced by improving the socio-economic position, this issue is among the main priorities in public health policies [1–3].

In Italy, equity in access to healthcare services, including medicines, is one of the main objective of the National Health Service (NHS) [4]. In this context, the “Essential Levels of Assistance” (LEA) delineate all the healthcare services that the NHS is required to provide to all Italian citizens (free of charge or upon a co-payment), regardless the individual ability to pay, income or region of residence, with the aim to ensure uniformity in healthcare assistance throughout the national territory [5]. In particular, LEAs also measure the appropriateness and the universality of territorial pharmaceutical care for people with chronic diseases [6].

However, despite the growing improvement in health conditions observed in recent years, there is still some evidence that the SEP is affecting the health status in Italy.

Moreover, wide differences persist also at geographical level: Southern Italy has more critical health indicators than other Italian areas. Such geographical variability depends on multiple factors; first of all the different organization of Regional Health Services (RHS), which could determine disparities in healthcare services and opportunities leading to an exacerbation of individual disadvantage of the lowest socioeconomic groups [7–13]. It’s known that drug use can be considered as a proxy indicator of chronic diseases and consequently of health status [14].

With regard to the Italian pharmaceutical assistance, few studies examined the role of socioeconomic factors in both access and optimal assumption of medicines; they were limited to single therapeutic categories or specific geographical areas and led to inconsistent results.

For example, a cohort study of about 70 thousand patients living in the city of Milan shows that, compared with people with the highest income, those with the lowest income were more likely to start antihypertensive and antidiabetic drug therapy; another study involving a total of 175 Italian health districts (3.3 million children/adolescents) found that lower income at the district level correlated with higher rates of antibiotic and anti-asthmatic drug prescriptions; similar results on antibiotic consumption are reported in a study of municipalities in a southern Italian region [15–18].

To date, no nationwide study, covering the majority of chronic diseases, has been performed to explore the correlation of medications use with the socioeconomic position of patients. The access to highest-quality medications for the management of both acute and chronic diseases, regardless of race and ethnicity, socio-economic position, or availability of resources, plays a decisive role for the quality of life. This goal could be referred as *pharmacoequity *[19].

The present study aims to compare the use of medicines – measured through consumption rate, adherence and persistence to drug therapy—prescribed for the major chronic diseases in Italy among groups of population characterized by a different level of SEP, measured through a *deprivation index* (DI). Such description of the phenomenon can be useful for national and regional interventions to be implemented in the country.

## Methods

### Data sources

A retrospective population-based study, considering all adult Italian residents (i.e. around 50.7 million people), was performed in order to compare the use of medicines prescribed for major chronic diseases in Italy among groups of population with different SEP. Data sources used to identify the study population were: the Italian pharmaceutical prescriptions database and the official registry of Italian resident population stratified by sex, age and municipality of residence for the year 2018 [20]. The pharmaceutical prescriptions database includes all individual records for each drug prescription dispensed by community pharmacies and reimbursed by the Italian NHS.

### Explanatory socio-economic variable

Considering the absence of socio-economic variables at individual level in pharmaceutical prescriptions database, a multidimensional measures of SEP defined as a national deprivation index (DI) was used to estimate the association with drugs use indicators. The DI used in this analysis is a composite measure, developed in 2010 [21] and further updated using data from 2011 census [22]; it takes into account five indicators representing material and social disadvantages: i) percentage of subjects with primary school education level or below, ii) percentage of the economically active subjects that is either unemployed or looking for a first job, iii) percentage of rented dwellings, iv) percentage of one parent families, and v) overcrowding (inhabitants per 100 m^2^ of living area). In order to study the relationship between SEP and drugs use, the Italian municipalities population were categorized by three tertiles of deprivation; the first tertile represented the least deprived group of population while the third tertile included the most deprived cases. Then, patients were assigned to each tertile on the basis of the DI referred to his municipality of residence. Given the high heterogeneity of distributions at the municipal level, to improve the readability of the results, the maps were represented by province, where each province was obtained from the weighted average of its constituent municipalities. Consistently, therefore, the DI map was also represented at the provincial level and divided into tertiles of deprivation. As shown from the progressive shade of colors, from lighter blue for provinces less deprived to dark blue for provinces more deprived, it can be observed how the DI is distributed differently along the Italian peninsula (eFigure 1).

### Chronic diseases and medications considered

Different medications were used as a proxy of underlying chronic diseases for all subjects aged ≥ 18 years: hypertension, dyslipidemia, osteoporosis, diabetes and chronic obstructive pulmonary disease (COPD). The medicines were classified according to the World Health Organization (WHO) Anatomical Therapeutic Chemical (ATC coding) classification system (see supporting information of table eTable 1 for further details on pharmacotherapy categories selected for the analysis) [23].

### Study designs and outcome indicators

Two observational study designs were considered to analyse the following three indicators on drugs use, medicine consumption rate, adherence and persistence to a pharmacological treatment:

- a cross-sectional design for the evaluation of consumption rate, which was used to define the *prevalent* users, i.e. all “*chronic*” patients who had a prescription for the selected pharmacological categories in the period from 1st January 2018 to 31st December 2018 (eTable 1);
- a longitudinal cohort design, to estimate one-year adherence and persistence among new drug users. To this aim, we considered as *incident* users all people who received at least one prescription in the period between 1st January 2018 and 31st December 2018 (*index period*) and who did not receive prescriptions for drugs belonging to the same therapeutical category in the previous 12 months (*wash-out period*). Subsequently, only incident users were followed for 365 days from the date of enrollment (index date) (see eTable 1).

The medicine consumption rate for each pharmacological category was defined as ratio between the total number of days of therapy (DOT) prescribed to patients and the residing population. DOT were calculated considering the *Daily Defined Dose* (DDD) associated to each prescribed medication.

The adherence to pharmacologic therapy was evaluated through the Medication Possession Ratio (MPR), calculated as the ratio of the amount of days a patient had his/her medicine on the time interval between first and last prescriptions. High-adherent patients were those with a number of days covered by pharmacologic therapy greater than 75% of the observed time period (i.e. MPR ≥ 75%) [24].

The persistence to pharmacologic therapy was defined as the time period between the initiation and the interruption of a prescribed drug treatment, considering as “*permissible gap*” an interruption of drug therapy shorter than 60 days [25].

### Statistical analysis

The medicine consumption rate was adjusted through a direct standardization, considering as standard population the official registry on Italian resident population for the year 2018 [20]. The adult population was stratified into 7 age groups (18–34 years; 35–44 years; 45–54 years; 55–64 years; 65–74 years; 75–84 years; ≥ 85 years).

The proportion of high adherent patients, adjusted for age and DI, was estimated through Poisson regression models. Persistence was estimated as the probability of therapeutic maintenance (“survival to the event”) after a fixed number of days from the beginning of therapeutic treatment [26]. In particular, the probability to be persistent after 12 months of treatment, adjusting for age and DI, was evaluated through the semi-parametric Cox regression model for survival analysis. The related 95% confidence intervals (CI) were calculated for each indicator.

Drug utilization analyses were stratified by tertile of deprivation. In a geographical analysis, the drug utilization indicators were represented on maps at provincial level and the DI was considered as a potential confounding variable of context and thus considered to adjust the outcomes indicators. In the graphical representation of each indicator the interquartile range (IQR) was reported. All results, adjusted for age, were calculated for both women and men.

For the graphical representation of the indicators at the provincial level, the existence of a spatial dependence among contiguous territorial areas was taken into account [27]. In fact, a Kernel estimator was used in order to obtain more reliable estimates in the proximity of the provincial boundaries to mitigate the variability related to this structural component of the phenomenon under consideration.

The statistical analyses were performed using SAS software, version 9.4 (SAS Institute, North Carolina, US).

## Results

**Fig. 1:**
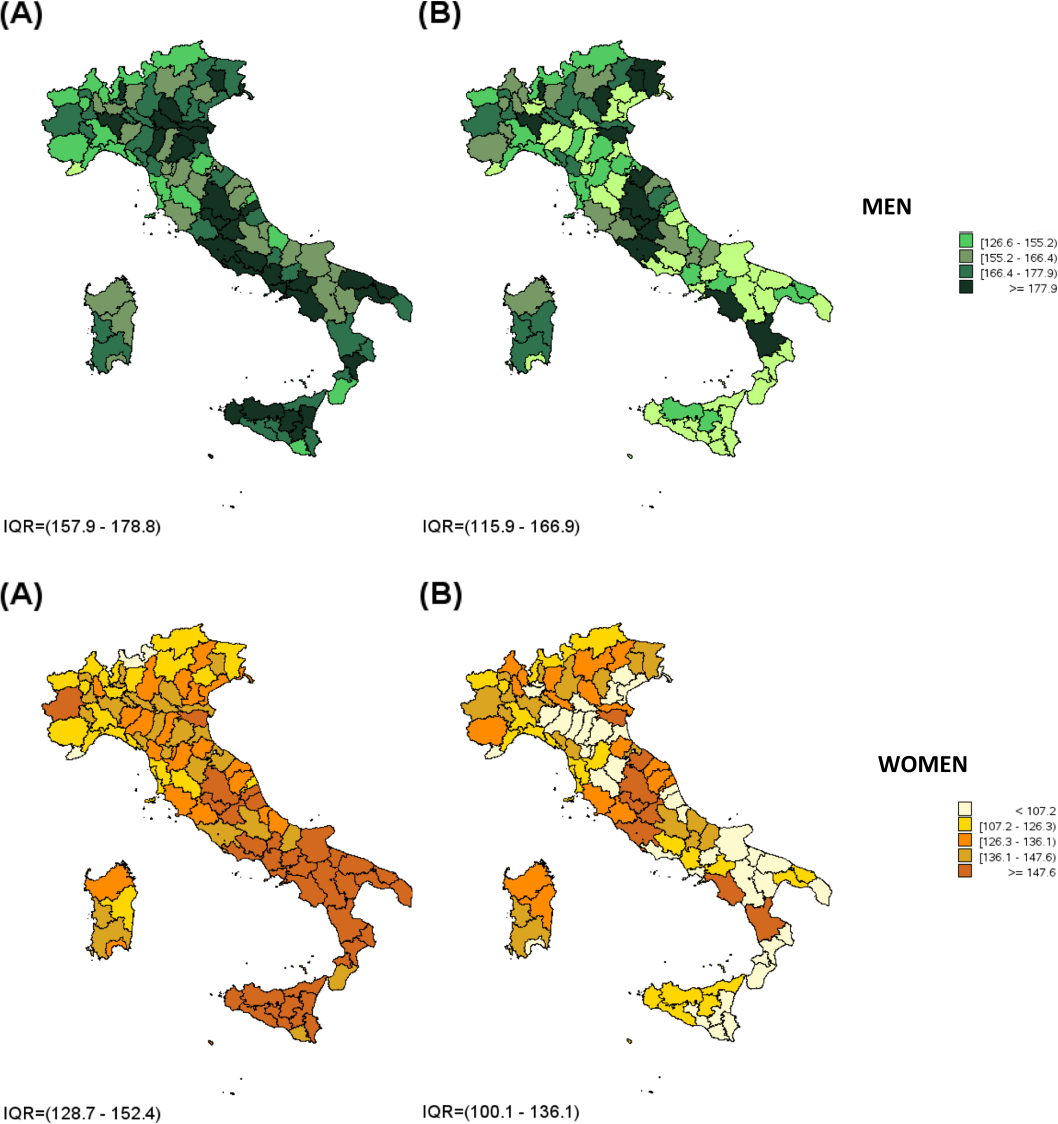
Medicine consumption rate (DDD per capita) for antihypertensive drugs in adults (aged ≥ 18 years) by province standardized: **A** by age only; **B** by age and deprivation tertile

**Fig. 2:**
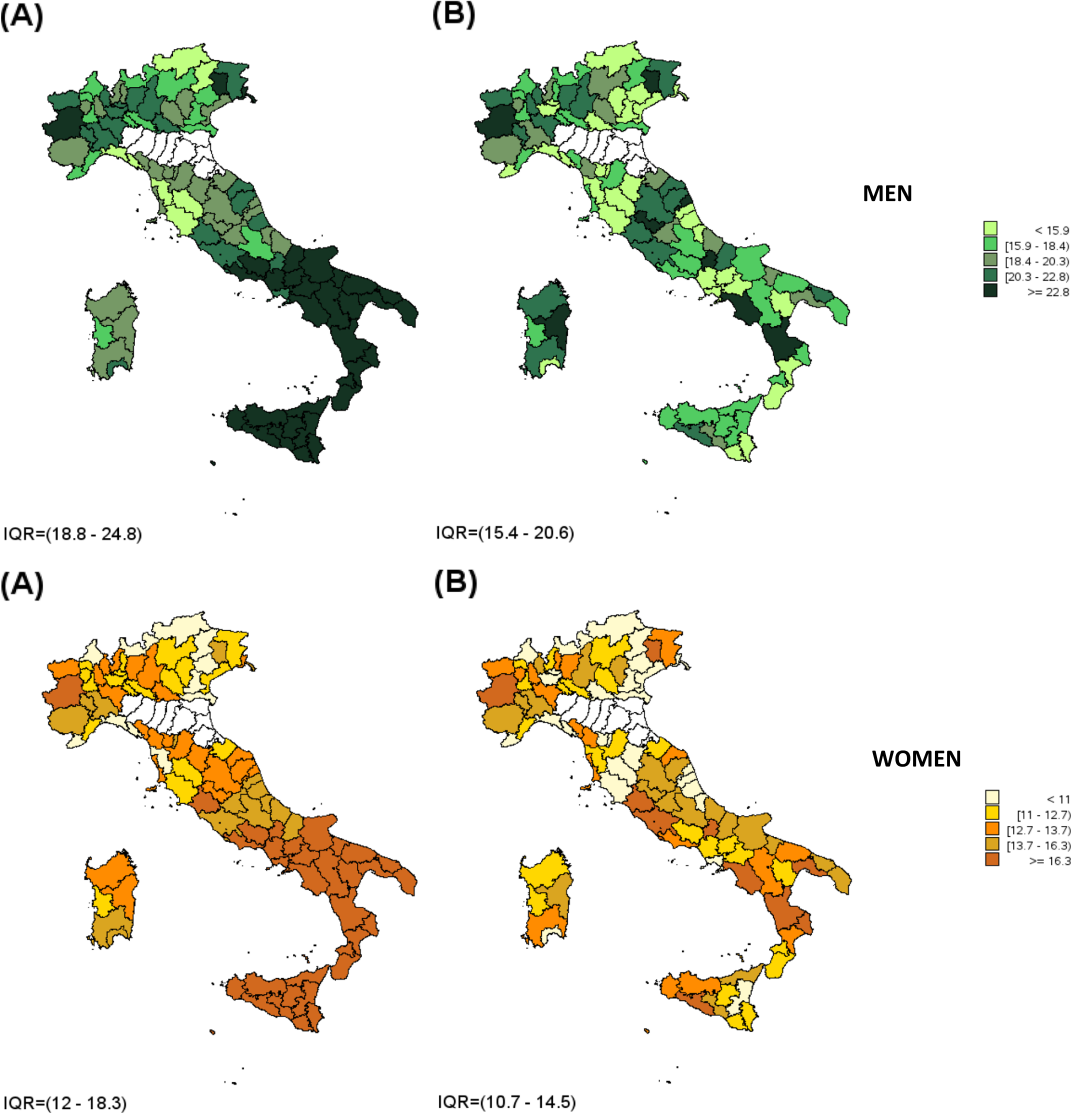
Medicine consumption rate (DDD per capita) for antidiabetics drugs in adults (≥ 18 years) by province† standardized: **A** by age only; **B** by age and deprivation tertile

Overall, higher levels of medication consumption rate were observed in men in comparison to women for all the selected therapeutic categories (170.8 DDD vs 141.8 DDD per capita for hypertension; 21.8 DDD vs 15.1 DDD per capita for diabetes; 44.3 DDD vs 30.0 DDD per capita for dyslipidemias; 9.9 DDD vs 6.5 DDD per capita for COPD) except for osteoporosis (0.6 DDD vs 6.6 DDD per capita). At the geographical level higher levels of consumption emerged in Southern Italy. The SEP was strongly correlated with medicine consumption rates, which were higher among subjects living in the most disadvantaged areas. This phenomenon was evident for almost all the chronic conditions analyzed, particularly for antihypertensive, hypolipidemic and antidiabetic medications as well as for COPD therapy, regardless of patient’s gender; similar results were observed for anti-osteoporotic medications in women. Removing the effect of deprivation, the medicines consumption decreased in the most deprived areas, mainly in Southern Italy (Table 1, Figs. 1 and 2, eFigures 2, 3 and 4).

**Table 1:** Medicine cconsumption rate in “prevalent” subjects treated for chronic diseases, standardized by age and stratified by gender and tertile of deprivation

		Men (≥ 18 year)			Women (≥ 18 year)		
	Tertiles of deprivation	Standardized consumption rate (95% CI)		Age-standardized consumption rate	Standardized consumption rate (95% CI)		Age-standardized consumption rate
**Hypertension**	1	165.30	(165.29–165.31)	170.85	128.10	(128.10–128.11)	141.78
	2	169.08	(169.07–169.09)		138.22	(138.21–138.22)	
	3	178.20	(178.19–178.21)		159.92	(159.91–159.93)	
**Dyslipidemia**	1	42.30	(42.30–42.31)	44.35	26.26	(26.26–26.26)	30.03
	2	43.14	(43.13–43.14)		28.70	(28.70–28.71)	
	3	47.68	(47.68–47.69)		35.42	(35.41–35.42)	
**Osteoporosis**	1	0.59	(0.59–0.59)	0.56	5.52	(5.52–5.53)	6.57
	2	0.56	(0.56–0.56)		6.32	(6.32–6.32)	
	3	0.51	(0.51–0.51)		7.97	(7.97–7.98)	
**Diabetes**	1	19.57	(19.57–19.58)	21.80	11.92	(11.92–11.93)	15.09
	2	20.52	(20.52–20.52)		13.95	(13.94–13.95)	
	3	25.00	(24.99–25.00)		19.07	(19.07–19.07)	
**COPD**	1	8.63	(8.63–8.63)	9.87	6.12	(6.12–6.12)	6.50
	2	9.50	(9.50–9.50)		6.48	(6.48–6.49)	
	3	11.42	(11.41–11.42)		6.89	(6.89–6.89)

**Fig. 3:**
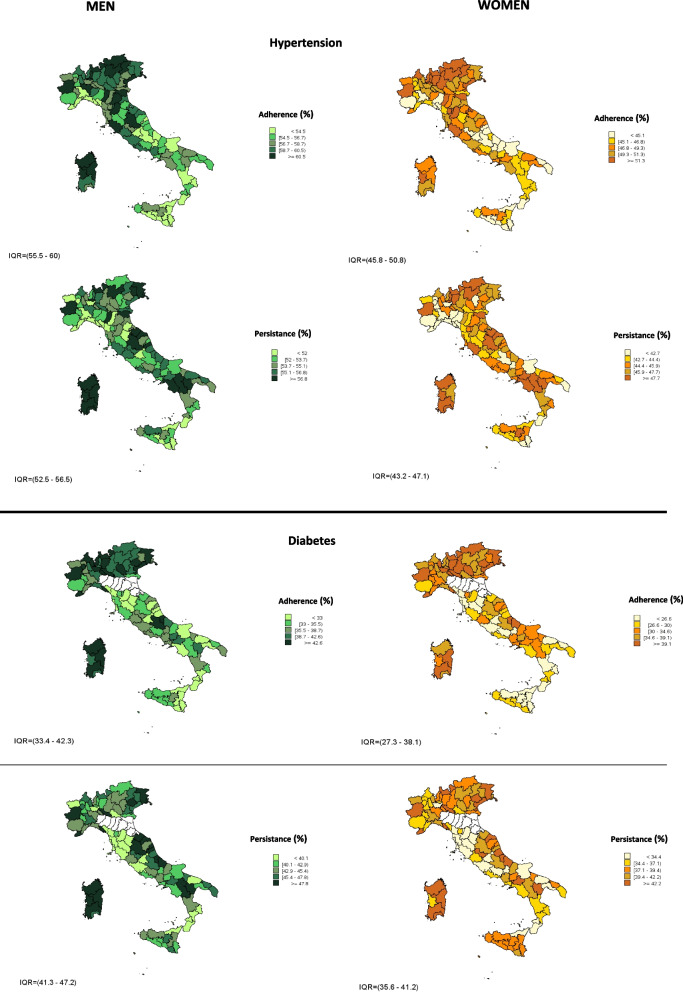
Adherence and persistence to treatment at 12 months (%) in adults (≥ 18 years) by province† adjusted by age

Adherence and persistence to the considered pharmacological treatments were found to be suboptimal at national level; moreover, a decreasing geographical North–South gradient was observed for both indicators. Women were less adherent to treatments than men (57.8% vs 48.5% for hypertension; 37.6% vs 31.6% for diabetes; 51.9% vs 40.5% for dyslipidemias; 36.1% vs 30.5% for COPD). The only exception was observed for anti-osteoporotic medications (close to 70% for both women and men). Levels of persistence to treatments observed for women were lower than men (54.2% in men and 45.0% for hypertension; 43.2% vs 36.8% for diabetes; 51.8% vs 43.4% for dyslipidemias; 51.8% vs 43.4% for COPD). By contrast, persistence at 12 months for anti-osteoporotic medications was 51.8% in women and 43.4% in men. With regard to the relationship with SEP at national level, both adherence and persistence were generally higher among people living in more deprived areas, however, a wide variability was observed within the different regions: a clear trend among different tertiles of deprivation was not observed in none of the Italian Regions. Removing the effect of deprivation, adherence and persistence levels remained constant, suggesting that the effect of deprivation is not appreciable in this context (Table 2, Fig. 3, eFigure 5).

**Table 2:** Adherence and persistence at 12 months (%) in “incident” subjects following a pharmacological treatment for chronic diseases, adjusted by age and stratified by gender and tertile of deprivation

		**Tertiles of deprivation**	**Incidents (n)**	**Adherence (%)**^a^	**Adherence adjusted for age %**	**Adherence adjusted for age and DI %**	**Relative difference between Adherence adjusted for age and adjusted for age and DI**	**Persistance (%)**^b^	**Persistance adjusted for age %**	**Persistance %**^**2**^	**Relative difference between Persistance adjusted for age and adjusted for age and DI**
**Hypertension**	**Men (≥ 18 year)**	1	152,364	58.7 (56,9–60,6)	57.8	57.9	0.1%	53.4 (53,1–53,6)	54.2	54.2	0.0%
		2	160,629	58.4 (56,6–60,2)				54.6 (54,4–54,8)			
		3	177,145	56.5 (54,9–58,3)				54.4 (54,2–54,7)			
	**Women (≥ 18 year)**	1	159,560	49.5 (47,9–51,1)	48.5	48.5	0.1%	44.1 (43,9–44,4)	45.0	45.0	0.0%
		2	167,975	49.2 (47,7–50,8)				45.5 (45,3–45,7)			
		3	176,907	47.0 (45,5–48,5)				45.4 (45,2–45,6)			
**Dyslipidemia**	**Men (≥ 18 year)**	1	103,990	53.8 (52,8–54,8)	51.9	52.1	0.4%	53.8 (53,5–54,1)	51.8	51.7	-0.1%
		2	112,564	52.7 (51,7–53,6)				52.8 (52,5–53,1)			
		3	137,595	50.0 (49,1–50,8)				49.3 (49,1–49,6)			
	**Women (≥ 18 year)**	1	103,113	42.3 (41,4–43,2)	40.5	40.8	0.6%	44.2 (43,9–44,5	43.4	43.4	0.1%
		2	114,359	41.3 (40,5–42,1)				44.2 (43,9–44,5)			
		3	146,235	38.8 (38,1–39,5)				42.1 (41,9–42,4)			
**Osteoporosis**	**Men (≥ 18 year)**	1	5,056	72.0 (69,6–74,4)	67.8	67.8	0.1%	49.5 (48,1–50,9)	43.4	43.0	-0.9%
		2	4,969	68.2 (65,9–70,5)				44.5 (43,2–45,9)			
		3	5,212	63.6 (61,5–65,8)				36.6 (35,3–37,9)			
	**Women (≥ 18 year)**	1	39,514	71.4 (70,0–73,0)	69.8	70.1	0.4%	55.4 (54,9–55,9)	51.8	51.9	0.2%
		2	44,308	70.7 (69,2–72,1)				53.0 (52,6–53,5)			
		3	54,808	68.2 (66,9–69,7)				48.3 (47,8–48,7)			
**Diabetes**	**Men (≥ 18 year)**	1	36,431	41.9 (39,9–43,9)	37.6	38.0	1.2%	44.0 (43,5–44,5)	43.2	43.2	0.0%
		2	43,054	37.4 (35,6–39,1)				42.7 (42,3–43,2)			
		3	54,985	35.0 (33,5–36,5)				43.1 (42,7–43,5)			
	**Women (≥ 18 year)**	1	29,196	37.1 (35,2–39,2)	31.6	32.2	1.8%	38.3 (37,8–38,9)	36.8	36.8	0.0%
		2	39,911	31.9 (30,4–33,6)				36.0 (35,5–36,4)			
		3	50,436	28.3 (27,0–29,7)				36.5 (36,1–37,0)			
**COPD**	**Men (≥ 18 year)**	1	40,013	37.1 (35,8–38,5)	36.1	36.4	0.8%	24.8 (24,4–25,3)	23.7	23.7	0.0%
		2	46,773	37.2 (36,0–38,5)				25.0 (24,6–25,4)			
		3	67,152	34.8 (33,8–35,8)				22.2 (21,9–22,5)			
	**Women (≥ 18 year)**	1	42,549	32.1 (30,9–33,3)	30.5	30.7	0.7%	19.9 (19,5–20,3)	18.0	18.0	-0.2%
		2	52,046	31.2 (30,2–32,3)				18.9 (18,6–19,3)			
		3	70,427	29.0 (28,2–29,9)				16.2 (15,9–16,5)			

^a^High adherence, calculated by MPR, was defined as therapeutic coverage ≥ 75% of the observation period (median (IQR): 284 (139–337)). The percentages were adjusted by age (see statistical methods for further details)

^b^Persistence to treatment was assessed in the 365 days following the first prescription (data index). An interruption to treatment occurs if the subject does not have a prescription delivered within 60 days (assessed on the basis of DDD). The percentages were adjusted by age (see statistical methods for more details)

A summary of the main evidence for each considered chronic disease is reported below. Tables 1 and 2 show the results for the five diseases, whereas the maps refer to the only two diseases with the highest prevalence rates (hypertension and diabetes).

## Discussion

This is the first study evaluating the relationship between SEP and several drug utilization indicators carried out in Italy at national level and covering the majority of chronic conditions. To our knowledge, previous studies were limited to single Italian regions or smaller samples [15–18].

Overall, the medicine consumption rates seem to be consistent with what is known on the epidemiology of chronic diseases in Italy and with the effects of deprivation on them. For example, the consumption rates of antihypertensive drugs are coherent with the results of several surveys conducted by Health Search, the research institute of the Italian Society of General Practitioners, which showed a higher prevalence of hypertension in the South of Italy than the Center and North and in poor subjects, compared to highly educated ones [28, 29]. A similar trend was also observed for the antidiabetic drugs [30, 31]. This aspect is more evident for antiosteoporotic drugs: since osteoporosis is a disease mainly affecting women, the consumption rate of these medications was 10 times higher in female than in male population [32]; only for dyslipidemias the medication consumption rates were lower in women than in men, despite the high prevalence of these disorders in both sex [29].

The results showed that SEP was strongly correlated with the use of medications, coherently with the social gradients in the occurrence of most diseases, suggesting that drugs are prescribed consistently with the health needs of the population. Therefore, they are in line with the “pharmacoequity” target expressed by the universality principle of the Italian NHS [19]. In Italy, in fact, the universal health system guarantees all people with free access to treatment, both in terms of drug consumption and access to the general practitioner.

High rates of consumption per capita were registered in areas characterized by high level of deprivation, in particular for therapeutic categories used to treat diabetes, hypertension, dyslipidemia, and, in women, for osteoporosis. An increasing trend of medicine consumption rates, from less to more deprived municipalities, was evident in almost all Italian regions. The adjustment by deprivation tertile sharply modified this trend causing a significantly reduction of consumption levels, especially in the most deprived provinces of Southern Italy thus reducing the difference between North and South.

This phenomenon could be probably explained by the worst health status of the subjects living in the poor areas, which could be associated with an unhealthy lifestyle. In fact, it is known that the medicine consumption rate is correlated with several factors, some are strictly related to the individual, such as the severity of the disease, awareness of health condition or compliance to therapy, and they may also be related to the socioeconomic level of individuals [8, 29]. Moreover, other factors associated with drug consumption are related to specific health care service characteristics, such as the different prescriptive behavior of medical doctors, and the different organization of local health services, including the access to general practitioners as well as the distribution of community pharmacies on the territories, which could explain part of consumption variability across geographical areas and among different Italian regions.

The average levels of adherence and persistence to pharmacological treatments were generally suboptimal; for both indicators a decreasing geographical North–South gradient was observed. However, the interpretation of the association with SEP was difficult because of the wide heterogeneity within the Italian regions and the lack of a univocal gradient: the national trend of deprivation tertiles, in fact, was not always coherent with the trend of intra-regional tertiles. On the contrary to what was observed in medicine consumption rates, where most of the variability disappeared after adjusting for deprivation, the levels of adherence and persistence did not change after removing the effect of deprivation, confirming the invariance of the two indicators with SEP. This result seems in contrast with a recent review that shows a positive impact of higher socioeconomic position on adherence for several chronic diseases, in particular for cardiovascular conditions [33]. However, other studies reported conflicting results and the evidence is not often conclusive [15, 34–43].

Adherence is a multifactorial phenomenon that can be affected by various factors both at individual and context level. Adherence to treatment for chronic diseases can be influenced by the territorial health care organization: for example, for diabetes, diagnostic-therapeutic care assistance is guaranteed by the presence of diabetes centres throughout the national territory. This organization, by increasing patient care, indirectly improves equity in access to pharmacological treatments [39, 40]. On the other hand, where therapeutic care is entrusted only to the general practitioner, individual characteristics and behaviours may play a key role on treatment compliance and the SEP indicator based on municipal area may not be suitable, suggesting the need to perform further studies using an individual SEP indicator.

So, as we reported above, the main healthcare challenge in Italy is to work hard on lifestyle and prevention starting from the most disadvantaged contexts in order to reduce inequities, rather than acting on access to therapies and treatments for chronicity.

The main limitation of the study is the lack of socio-economic indicators at individual level. The use of a municipal deprivation indicator necessarily generates an ecological bias, whose entity is difficult to quantify. This bias, moreover, may have a greater impact on adherence and persistence, not allowing to detect the potential impact of socio-economic determinants, which in these cases presumably act on an individual level (unlike what is observed for consumption rates, where contextual socio-economic factors captured by the aggregate index highlighted an impact). In order to evaluate the inclusion of wide geographical aggregated in the analysis, sensitivity analyses were conducted for all indicators, restricting the analysis to municipalities with less than 30,000 inhabitants, that is the threshold indicated for the validity of the use of the deprivation index [44]; the results showed no significant differences compared to the main analysis (results not shown).

A further limitation was the inability to trace medicines purchased privately by Italian citizens, which are not recorded by the pharmaceutical information system. Since the private purchase of drugs is higher for the wealthier subjects than for those more disadvantaged, it could lead to an overestimation of the large variability in the consumption rate observed for some therapeutic category.

Nevertheless, the study confirmed the analytical potential of pharmaceutical prescriptions databases: the population taken into analysis is in fact the whole adult Italian population (about 50.7 million people) for which the entire prescribing history was available along with socioeconomic information at the municipality level.

## Conclusion

Drug consumption is influenced by the level of deprivation consistently with the distribution of diseases. This phenomenon could be probably explained by the worst health status of the subjects living in the poor areas, which could be associated with an unhealthy lifestyle, so the main levers on which it is necessary to act to reduce disparities in health status are mainly related to prevention.

The nationwide comparative approach of health care-related phenomena is a fundamental stimulus to improve health assistance, from the central to the local level of government. The experience of the present study, which for the first-time deals with the complex and delicate issue of equity in pharmaceutical assistance, lays the groundwork for new insights that could overcome the limitations of the methodology used here and make a further contribution to the understanding of this phenomenon.

## Supplementary Information

**Additional file 1:eTable 1.** Therapeutic categories and exposure for single chronic disease. **eFigure 1.** Provincial deprivation index map. **eFigure 2.** Medicine consumption rate (DDD per capita) for lipid-lowering agent in adults (aged ≥ 18 years) by province, standardized: (**A**) by age only; (**B**) by age and deprivation tertile. **eFigure 3.** Medicine consumption rate (DDD per capita) for antiosteoporotic drugs in adults (aged ≥ 18 years) by province, standardized: (**A**) by age only; (**B**) by age and deprivation tertile. **eFigure 4.** Medicine consumption rate (DDD per capita) for drugs for obstructive airway diseases in adults (aged ≥ 18 years) by province†, standardized: (**A**) by age only; (**B**) by age and deprivation tertile. **eFigure 5.** Adherence and persistence to treatment at 12 months (%) in adults (≥ 18 years) by province adjusted by age.

## Data Availability

The data that support the findings of this study are available from the Italian Medicines Agency but restrictions apply to the availability of these data, which were used under license for the current study, and so are not publicly available. Data are however available from the authors upon reasonable request.

## References

[1] Mackenbach JP, Stirbu I, Roskam AJ (2008). European Union Working Group on Socioeconomic Inequalities in Health. Socioeconomic inequalities in health in 22 European countries. N Engl J Med.

[2] World Health Organization (2010). A conceptual framework for action on the social determinants of health. Social Determinants of health, discussion paper 2.

[3] European Commission, Directorate-General for Health and Consumers, Meerding, W., Kunst, A., Mackenbach, J. Economic implications of socio-economic inequalities in health in the European Union, European Commission. 2010 https://op.europa.eu/en/publication-detail/-/publication/759fa7f6-a24c-46cc-acbe-98280eed55fc. Accessed 15 June 2022

[4] Art.1 LEGGE 23 dicembre 1978, n. 833, GU Serie Generale n.360 del 28–12–1978 - Suppl. Ordinario. https://www.gazzettaufficiale.it/eli/id/1978/12/28/078U0833/sg. Accessed 15 June 2022

[5] Decreto del presidente del consiglio dei ministri 12 gennaio 2017. Definizione e aggiornamento dei livelli essenziali di assistenza, di cui all'articolo 1, comma 7, del decreto legislativo 30 dicembre 1992, n. 502, G.U. Serie Generale , n. 65 del 18 marzo 2017. https://www.trovanorme.salute.gov.it/norme/dettaglioAtto?id=58669&completo=false. Accessed 15 June 2022

[6] Interministerial Decree March 12, 2019 "New guarantee system for health care monitoring" Art.3, paragraph 1. https://www.salute.gov.it/imgs/C_17_pagineAree_5238_2_file.pdf. Accessed 12 Sept 2022

[7] Costa G., Bassi M., Gensini G.F., Marra M., Nicelli A.L., Zengarini N. L’equità nella salute in Italia- Secondo rapporto sulle disuguaglianze sociali in sanità. Milano: Franco Angeli e Fondazione Smith Kline; 2014,188-223.

[8] Petrelli A, Di Napoli A, Sebastiani G (2019). Italian Atlas of mortality inequalities by education level. Epidemiol Prev.

[9] De Curtis M, Bortolan F, Diliberto D, Villani L (2021). Pediatric interregional healthcare mobility in Italy. Ital J Pediatr.

[10] Landi S, Ivaldi E, Testi A (2021). The role of regional health systems on the waiting time inequalities in health care services: Evidences from Italy. Health Serv Manage Res.

[11] Lallo C, Raitano M (2018). Life expectancy inequalities in the elderly by socioeconomic status: evidence from Italy. Popul Health Metr.

[12] Landi S, Ivaldi E, Testi A (2018). Socioeconomic status and waiting times for health services: An international literature review and evidence from the Italian National Health System. Health Policy.

[13] de Waure C, Bruno S, Furia G, Di Sciullo L, Carovillano S, Specchia ML, Geraci S, Ricciardi W (2015). Health inequalities: an analysis of hospitalizations with respect to migrant status, gender and geographical area. BMC Int Health Hum Rights.

[14] Kasper JA, Wilson R (1983). Use of prescribed medicines: a proxy indicator of access and health status. Int J Health Serv.

[15] Corrao G, Zambon A, Parodi A, Mezzanzanica M, Merlino L, Cesana G, Mancia G (2009). Do socioeconomic disparities affect accessing and keeping antihypertensive drug therapy? Evidence from an Italian population-based study. J Hum Hypertens.

[16] Kirchmayer U, Agabiti N, Belleudi V (2012). Socio-demographic differences in adherence to evidence-based drug therapy after hospital discharge from acute myocardial infarction: a population-based cohort study in Rome. Italy J Clin Pharm Ther.

[17] Piovani D, Clavenna A, Cartabia M, Bonati M (2014). Interregional Italian Drug Utilisation Group. Antibiotic and anti-asthmatic drug prescriptions in Italy: geographic patterns and socioeconomic determinants at the district level. Eur J Clin Pharmacol.

[18] Russo V, Monetti VM, Guerriero F (2018). Prevalence of antibiotic prescription in southern Italian outpatients: real-world data analysis of socioeconomic and sociodemographic variables at a municipality level. Clinicoecon Outcomes Res.

[19] Essien UR, Dusetzina SB, Gellad WF (2021). A Policy Prescription for Reducing Health Disparities - Achieving Pharmacoequity. JAMA.

[20] ISTAT. http://dati-censimentipermanenti.istat.it/. Accessed June 15, 2022.

[21] Caranci N, Biggeri A, Grisotto L, Pacelli B, Spadea T, Costa G (2010). The Italian deprivation index at census block level: definition, description and association with general mortality. Epidemiol Prev.

[22] Rosano A, Pacelli B, Zengarini N, Costa G, Cislaghi C, Caranci N (2020). Aggiornamento e revisione dell’indice di deprivazione italiano 2011 a livello di sezione di censimento. Epidemiol Prev.

[23] WHO Collaborating Centre for Drug Statistics Methodology. Anatomical Therapeutic Chemical (ATC) index with Defined Daily Doses (DDDs) 2020. www.whocc.no/atc_ddd_index/. Accessed 15 June 2022

[24] Di Martino M, Alagna M, Cappai G (2016). Adherence to evidence-based drug therapies after myocardial infarction: is geographic variation related to hospital of discharge or primary care providers? A cross-classified multilevel design. BMJ Open.

[25] Santoni L, Dall’ Asta G, Spampinato A (2009). Aderenza e persistenza alla terapia con statine: analisi di farmacoutilizzazione a partire dai database amministrativi di cinque ASL italiane. Giornale Italiano di Farmacoeconomia e Farmacoutilizzazione.

[26] Rasmussen L, Pratt N, Hansen MR, Hallas J, Pottegård A (2018). Using the “proportion of patients covered” and the Kaplan- Meier survival analysis to describe treatment persistence. Pharmacoepidemiol Drug Saf.

[27] Anselin L (1995). Local Indicators of Spatial Association–LISA. Geogr Anal.

[28] Osservatorio Nazionale sull’impiego dei Medicinali. L’uso dei Farmaci in Italia. Rapporto Nazionale Anno 2020. Roma: Agenzia Italiana del Farmaco (AIFA), 2021.

[29] Il Progetto Cuore - Epidemiologia e prevenzione delle malattie cerebro e cardiovascolari. http://www.cuore.iss.it/. Accessed 15 June 2022.

[30] Gnavi R, Migliardi A, Maggini M, Costa G (2018). Prevalence of and secular trends in diagnosed diabetes in Italy: 1980–2013. Nutr Metab Cardiovasc Dis.

[31] Minardi V, Ferrante G, Possenti V (2011). I numeri di PASSI. Anche i dati di sorveglianza confermano: il diabete è associato allo svantaggio economico-sociale. Epidemiol Prev.

[32] ISTAT. Multiscopo sulle famiglie: aspetti della vita quotidiana - parte generale. Anno 2018. https://www4.istat.it/it/archivio/91926. Accessed 15 June 2022.

[33] Gast A, Mathes T (2019). Medication adherence influencing factors—an (updated) overview of systematic reviews. Syst Rev.

[34] Gulizia MM, Colivicchi F, Ricciardi G (2017). Joint Consensus Document on cholesterol and cardiovascular risk: diagnostic-therapeutic pathway in Italy et al sl. Eur Heart J Suppl.

[35] Alsabbagh MH, Lemstra M, Eurich D (2014). Socioeconomic status and nonadherence to antihypertensive drugs: a systematic review and meta-analysis. Value Health.

[36] Vallée A, Grave C, Gabet A, Blacher J, Olié V (2021). Treatment and adherence to antihypertensive therapy in France: the roles of socioeconomic factors and primary care medicine in the ESTEBAN survey. Hypertens Res.

[37] Yeam CT, Chia S, Tan HCC, Kwan YH, Fong W, Seng JJB (2018). A systematic review of factors affecting medication adherence among patients with osteoporosis. Osteoporosis Int.

[38] Pepe J, Cipriani C, Cecchetti V (2019). Patients’ reasons for adhering to long-term alendronate therapy. Osteoporos Int.

[39] Gnavi R, Picariello R, Pilutti S, Di Monaco R, Oleandri S, Costa G (2020). Epidemiology in support of intervention priorities: the case of diabetes in Turin (Piedmont Region) s.l. Epidemiol Prev.

[40] Bartolini L, Caranci N, Gnavi R, Di Girolamo C (2020). Educational inequalities in the prevalence and outcomes of diabetes in the Emilian longitudinal study. Nutr Metab Cardiovasc Dis.

[41] Espelt A, Arriola L, Borrell C, Larrañaga I, Sandín M, Escolar-Pujolar A (2011). Socioeconomic position and type 2 diabetes mellitus in Europe 1999–2009: a panorama of inequalities. Curr Diabetes Rev.

[42] Tøttenborg SS, Lange P, Johnsen SP, Nielsen H, Ingebrigtsen TS, Thomsen RW (2016). Socioeconomic inequalities in adherence to inhaled maintenance medications and clinical prognosis of COPD. Respir Med.

[43] Price D, Keininger DL, Viswanad B, Gasser M, Walda S, Gutzwiller FS (2018). Factors associated with appropriate inhaler use in patients with COPD - lessons from the REAL survey. Int J Chron Obstruct Pulmon Dis.

[44] Pasetto R, Caranci N, Pirastu R (2011). Deprivation indices in small area studies of environment and health in Italy. Epidemiol Prev.

