# Supplementary material for: Socio-economic inequalities in the use of drugs for the treatment of chronic diseases in Italy
Source: Int J Equity Health. 2022 Nov 9;21:157. doi: 10.1186/s12939-022-01772-8 (PMC9644599; doi:10.1186/s12939-022-01772-8)
Supplement: Supplementary file 1 — Additional file 1:eTable 1. Therapeutic categories and exposure for single chronic disease. eFigure 1. Provincial deprivation index map. eFigure 2. Medicine consumption rate (DDD per capita) for lipid-lowering agent in adults (aged ≥ 18 years) by province, standardized: (A) by age only; (B) by age and deprivation tertile. eFigure 3. Medicine consumption rate (DDD per capita) for antiosteoporotic drugs in adults (aged ≥ 18 years) by province, standardized: (A) by age only; (B) by age and deprivation tertile. eFigure 4. Medicine consumption rate (DDD per capita) for drugs for obstructive airway diseases in adults (aged ≥ 18 years) by province†, standardized: (A) by age only; (B) by age and deprivation tertile. eFigure 5. Adherence and persistence to treatment at 12 months (%) in adults (≥ 18 years) by province adjusted by age. [file 12939_2022_1772_MOESM1_ESM.docx]

## Supplementary files

**Socio-economic inequalities in the use of drugs for the treatment of chronic diseases in Italy.**

**A. Di Filippo^1^**^†󠅟^**, S. Perna^1^**^†󠅟^**, A. Pierantozzi^1^, F. Milozzi^1^, F. Fortinguerra^1^, N. Caranci^2^, L. Moro^2^, N. Agabiti^3^, V. Belleudi^3^, G. Cesaroni^3^, A. Nardi^3^, T. Spadea^4^, R. Gnavi^4^, F. Trotta^1^**

**^1^** Italian Medicines Agency (AIFA), Rome, Italy.

^2^ Regional Health and Social Care Agency, Emilia-Romagna Region, Bologna, Italy

^3^ Department of Epidemiology, Lazio Regional Health Service, Rome, Italy

^4^ Epidemiology Unit ASL TO3, Piedmont Region, Turin, Italy

†󠅟 These authors contributed equally to this work and share first authorship

**Drug Inequalities study group:** Silvia Miriam Cammarata, Aurora Di Filippo, Filomena Fortinguerra, Nicola Magrini, Federica Milozzi, Serena Perna, Andrea Pierantozzi, Giuseppe Traversa, Francesco Trotta (Italian Medicines Agency, Rome); Antonio Addis, Nera Agabiti, Anna Maria Bargagli, Valeria Belleudi, Enrico Calandrini, Silvia Cascini, Giulia Cesaroni, Marina Davoli, Angelo Nardi (Department of Epidemiology, Lazio Regional Health Service, ASL Roma 1, Rome); Nicola Caranci, Maria Luisa Moro (Regional Health and Social Care Agency, Emilia-Romagna Region, Bologna, Italy); Roberto Gnavi, Teresa Spadea (Epidemiology Unit ASL TO3, Piedmont Region, Turin, Italy)

**Tables:** 1

**Figures:** 5

Corresponding author:

Aurora Di Filippo

Italian Medicines Agency (AIFA)

Via del Tritone 181 – 00187 Roma

Email:a.difilippo@aifa.gov.it

### eTable 1 -Therapeutic categories and exposure for single chronic disease.

| **Chronic disease** | **Pharmacotherapy category (*italic*), pharmaceutical subgroups (•) and drugs (•)** | **Subjects in therapy for chronic disease** |
| --- | --- | --- |
| **Hypertension** | ***Antihypertensive drug:***   - Antihypertensives (ATC: C02) - Diuretics (ATC: C03) - Beta blocking agents (ATC: C07) - Calcium channel blockers ATC: C08) - Agents acting on the renin–angiotensin system (ATC: C09) | Patients with at least 2 prescriptions within the same subgroup of drugs during the year |
| **Dyslipidemias** | ***Lipid-lowering agent:***   - Fibrates (ATC: C10AB) - HMG CoA reductase inhibitors (ATC: C10AA) - Other lipid modifying agents (ATC: C10AX)   [Ezetimibe](https://it.wikipedia.org/wiki/Ezetimibe) (ATC: C10AX09)   - Lipid modifying agents, combinations (ATC: C10B) | Patients with at least 2 prescriptions within the same subgroup of drugs or specific medicine during the year |
| **Osteporosis** | ***Antiosteoporotic drugs***   - Selective estrogen receptor modulators (ATC:G03XC)   - - - Raloxifene (ATC: G03XC01)       - Bazedoxifene (ATC: G03XC02) - Bisphosphonates (ATC: M05BA) - Bisphosphonates, combinations (ATC: M05BB) - Parathyroid hormones and analogues   Teriparatide (ATC H05AA02) | Patients with at least 2 prescriptions within the same subgroup of drugs or specific medicine during the year |
| **Diabetes** | ***Drugs used in diabetes (non-insulin therapy for type 2 diabetes)***   - Biguanides (ATC: A10BA)   - - - Metformin (ATC A10BA02) - Combinations of oral blood glucose lowering drugs (ATC: A10BD) - metformin + pioglitazone(ATC: A10BD05) - metformin + sitagliptin (ATC A10BD07) - metformin + ildagliptin (ATC A10BD08) - metformin + saxagliptin (ATC A10BD10) - metformin + linagliptin (ATC A10BD11) - metformin + alogliptin (ATCA10BD13) - metformin + dapagliflozin (ATCA10BD15) - metformin + canagliflozin(ATC A10BD16)   - - - metformin + empagliflozin(ATC A10BD20) | Patients with at least 2 prescriptions within the same subgroup of drugs or specific medicine during the year Please note that for the subjects included in the analyses, the indicators were calculated considering the exposure to all pharmacotherapy category A10. |
| **Chronic Obstructive Pulmonary Disease (COPD)** | ***Drugs for obstructive airway diseases***   - Monotherapy of LABA (ATC: R03AC) - salmeterol (ATC: R03AC12) - sormoterol (ATC: R03AC13) - indacaterol (ATC: R03AC18) - olodaterol (ATC: R03AC19) - Monotherapy of LAMA (ATC: R03BB) - tiotropium bromide (ATC: R03BB04) - aclidinium bromide (ATC: R03BB05) - glycopyrronium bromide (ATC: R03BB06) - umeclidinium bromide (ATC: R03BB07) - LABA + ICS (ATC: R03AK) - Salmeterol + fluticasone (ATC: R03AK06) - formoterol + budesonide (ATC: R03AK07) - formoterol + beclometasone (ATC: R03AK08) - vilanterol + fluticasone (ATC: R03AK10) - formoterol + fluticasone (ATC: R03AK11) - formoterolo+ mometasone (ATC: R03AK09) - LABA + LAMA (ATC: R03AL) - vilanterol + umeclidinium bromide (ATC: R03AL03) - indacaterol + glycopyrronium bromide (ATC: R03AL04) - formoterol and aclidinium bromide (ATC: R03AL05) - olodaterol and tiotropium bromide (ATC: R03AL06) - LABA + LAMA + ICS   formoterol, glycopyrronium bromide and beclometasone (ATC: R03AL09) | Patients with at least 2 prescriptions within the same subgroup of drugs during the year (monotherapy of LABA or monotherapy of LAMA or LABA+ICS or LABA+LAMA+ICS) |

### eFigure 1. Provincial deprivation index map


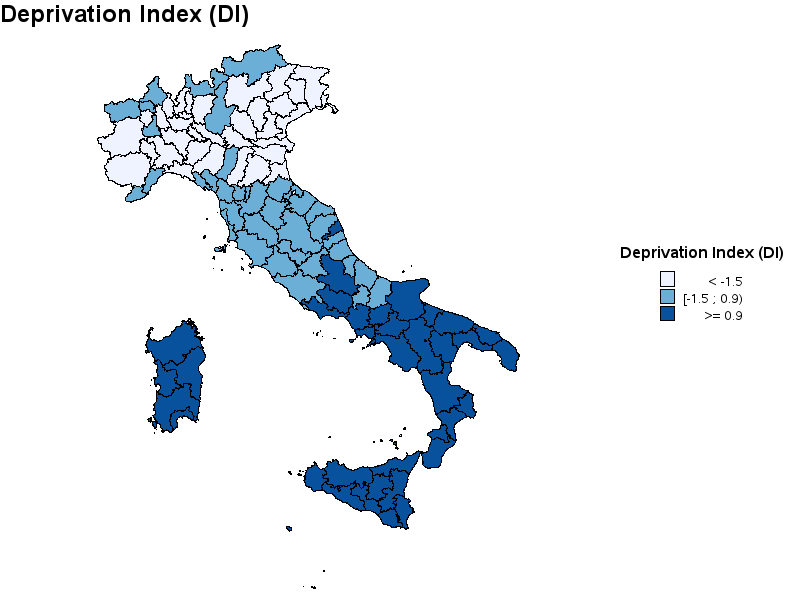


### eFigure 2: Medicine consumption rate (DDD per capita) for lipid-lowering agent in adults (aged ≥ 18 years) by province, standardized: (A) by age only; (B) by age and deprivation tertile


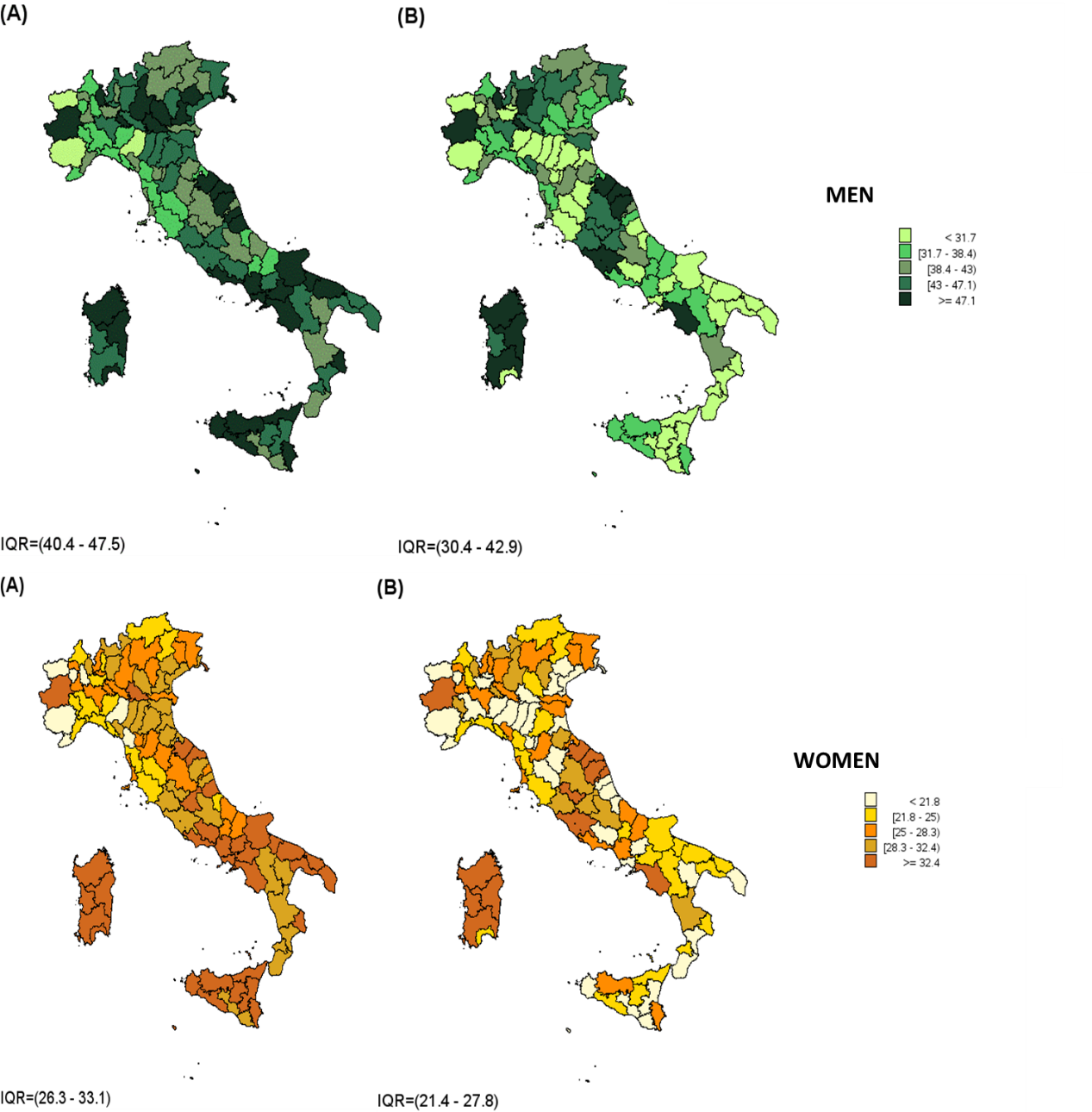


### eFigure 3: Medicine consumption rate (DDD per capita) for antiosteoporotic drugs in adults (aged ≥ 18 years) by province, standardized: (A) by age only; (B) by age and deprivation tertile

**
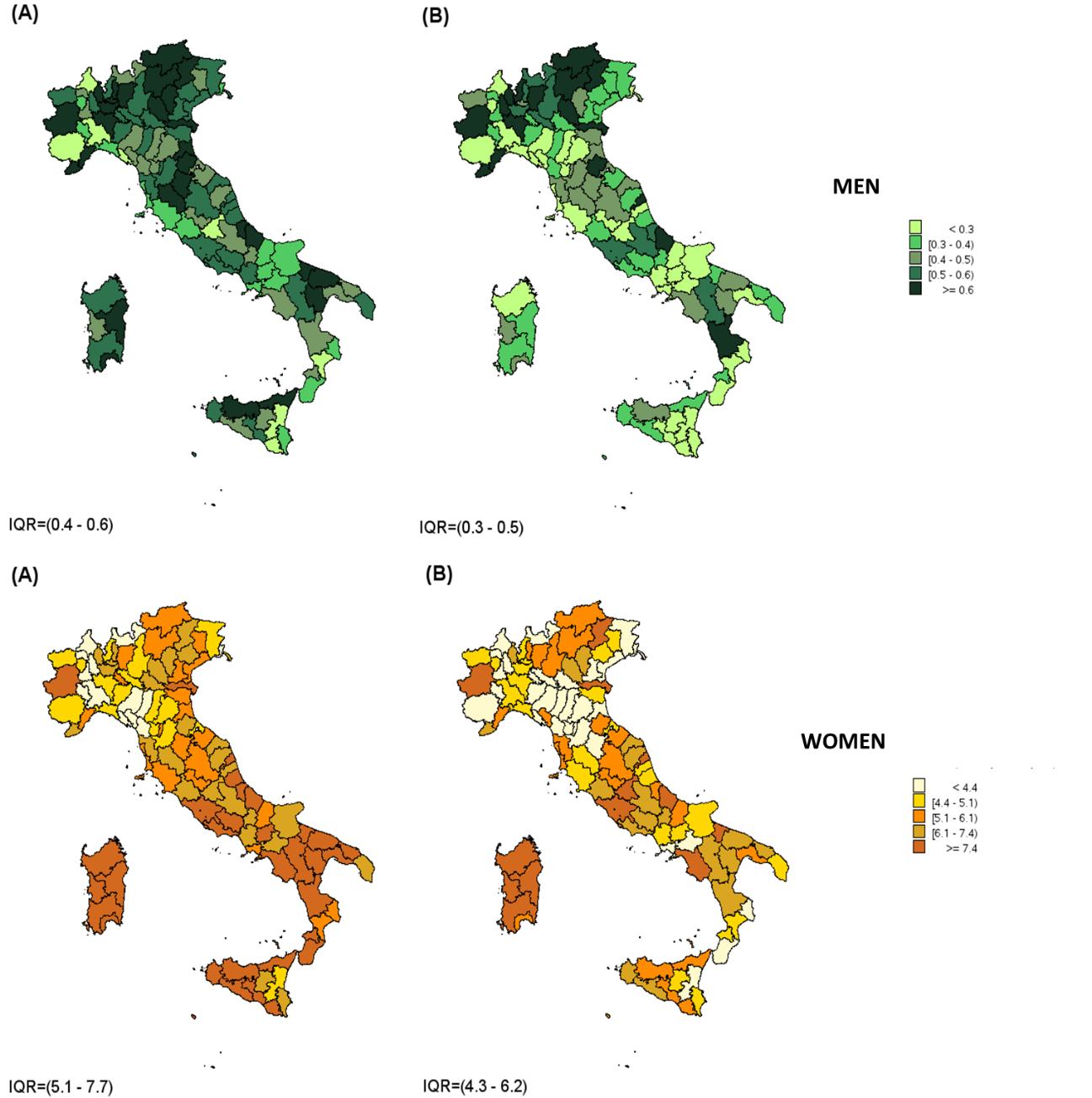
**

### eFigure 4: Medicine consumption rate (DDD per capita) for drugs for obstructive airway diseases in adults (aged ≥ 18 years) by province†, standardized: (A) by age only; (B) by age and deprivation tertile

**
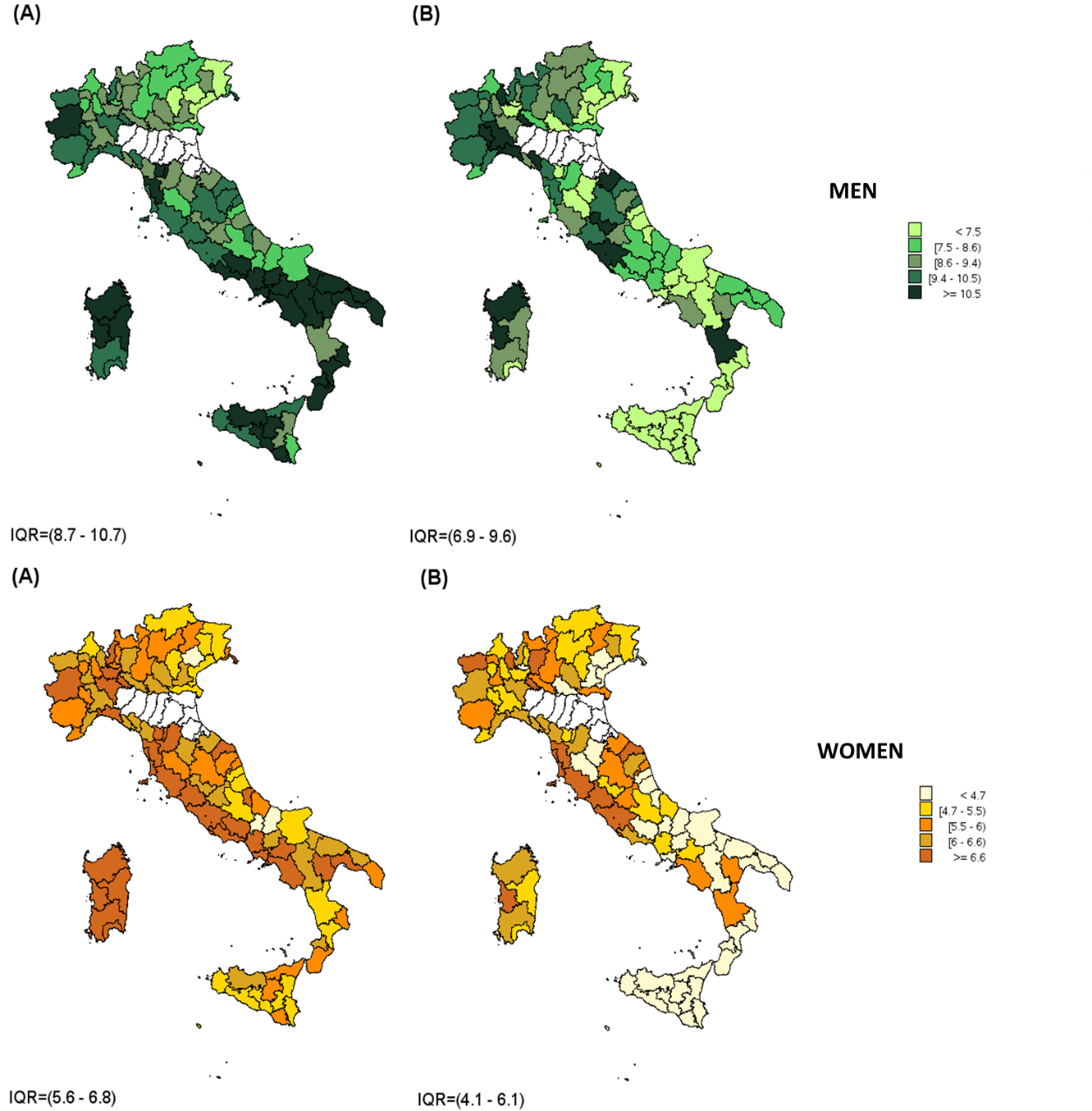
**

†Italian regions for which the percentage of drugs dispended under the territorial assistance system (including DPC distribution) was less than 85% of the total quantity dispensed were excluded from the analysis (blank in the map)

### eFigure 5: Adherence and persistence to treatment at 12 months (%) in adults (≥ 18 years) by province adjusted by age

**
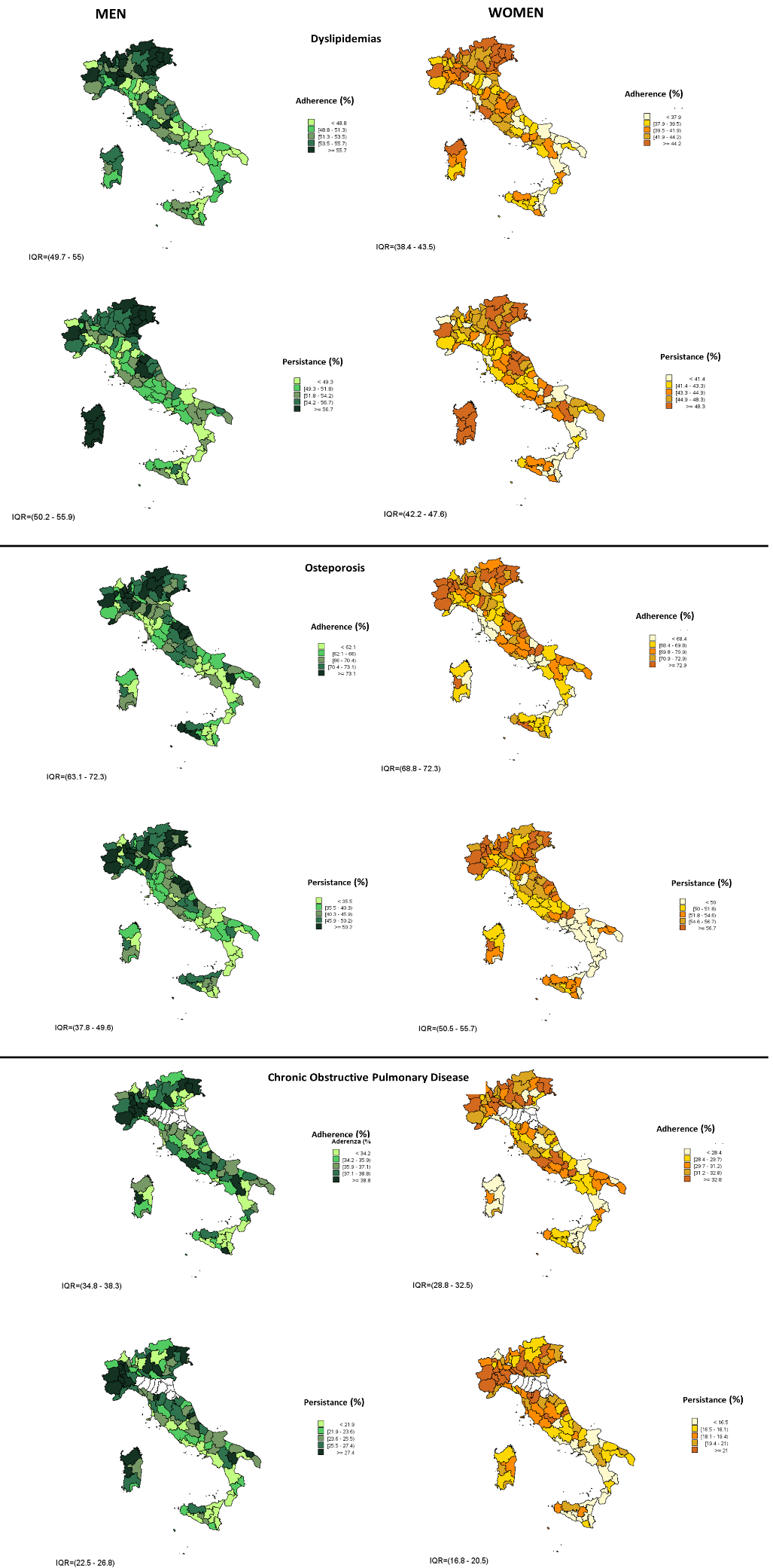
**

† Italian regions for which the percentage of drugs dispended under the territorial assistance system (including DPC distribution) was less than 85% of the total quantity dispensed were excluded from the analysis (blank in the map)
